# Simulation of Residual Stress Around Nano-Perforations in Elastic Media: Insights for Porous Material Design

**DOI:** 10.3390/ma18235388

**Published:** 2025-11-29

**Authors:** Shuang Wang, Xin Jia, Kun Song, Haibing Yang, Shichao Xing, Hongyuan Li, Ming Cheng

**Affiliations:** 1 School of Physical and Mathematical Sciences, Nanjing Tech University, Nanjing 211816, China; shuangwang@njtech.edu.cn (S.W.);; 2 School of Mechanical and Power Engineering, Nanjing Tech University, Nanjing 211816, China; 3 College of Mechanics and Engineering Science, Hohai University, Nanjing 211100, China; 4 Anhui Joint Key Laboratory of Critical Technologies for High-End Copper-Based New Materials, Tongling University, Tongling 244061, China; 5 Wan Jiang New Industry Technology Development Center, Tongling 244031, China; 6 State Key Laboratory for Turbulence and Complex Systems, Department of Mechanics and Engineering Science, BIC-ESAT, College of Engineering, Peking University, Beijing 100871, China; 7 Peking University Nanchang Innovation Institute, Nanchang 330096, China

**Keywords:** nano-perforation, porous material, surface tension, residual stress, complex variable method, stress concentration

## Abstract

The mechanical integrity of advanced porous materials and perforated structures at the nanoscale is critically governed by the interaction of surface effects and stress concentration around pore architectures. This paper investigates the residual stress field induced by surface tension around two arbitrarily shaped nano-perforations within an infinite elastic matrix, a configuration highly relevant to nanoporous metals and functional composites. By leveraging the complex variable method and conformal mapping techniques, the physical domains of the perforations (approximated as triangular and square shapes, paired with an elliptical perforation) are transformed into unit circles. This transformation allows for the derivation of semi-analytical solutions for the complex potentials and the subsequent stress field. Systematic numerical case studies reveal that a reduced inter-perforation distance dramatically intensifies the hoop stress concentration at the adjacent vertices, identifying these sites as potential initiation points for mechanical failure. Conversely, an increase in the size of one perforation can effectively shield its neighbor and reduce the overall stress level. These findings provide quantitative, physics-based guidelines for the microstructural design of nanoporous materials. By consciously tailoring the spatial distribution, size, and shape of perforations, the mechanical reliability of nanomaterials can be rationally optimized for applications in nanoscale systems.

## 1. Introduction

The advent of nanotechnology has propelled the development of advanced porous materials, such as nanoporous gold, metal–organic frameworks (MOFs), and mesoporous silica, which exhibit exceptional properties for applications in catalysis, energy storage, and sensing [1,2,3]. Similarly, perforated structures with nanoscale features are fundamental to the design of micro- and nano-electromechanical systems (MEMS/NEMS), photonic crystals, and composite materials [4,5,6]. A common critical factor determining the performance and reliability of these materials and devices is their mechanical behavior, which is dominated by stress concentrations around the pore architectures [6,7].

At the nanoscale, surface effects, often negligible in their macroscopic counterparts, become paramount. The Gurtin-Murdoch (G-M) continuum surface elasticity model [8,9,10] provides a robust framework for incorporating these effects, treating the surface as a thin membrane with its own elastic properties and a constant residual surface tension. The residual surface tension, even in the absence of external loads, induces a self-equilibrated stress field that is proportional to the local curvature at the perforation boundary [11]. This phenomenon can significantly alter the effective elastic properties and failure mechanisms of nanoscale porous and perforated materials [12,13].

Extensive research has been dedicated to understanding the elastic fields and effective mechanical properties of nanocomposites containing a single nano-perforation or inclusion. Seminal works have provided analytical and semi-analytical solutions for circular and elliptical inclusions [14,15,16,17,18], which were later extended to stress analysis around an elliptical nano-perforation [18] and later to perforations of arbitrary shape [11,19], revealing that hoop stress can attain minima at sharp vertices.

However, the practical reality of materials science involves a multitude of pores and heterogeneities. The mechanical interaction between multiple nanoscale perforations remains largely uncharted territory, with existing studies predominantly limited to analytical or semi-analytical solutions to circular or elliptical perforations [20,21]. The interaction between non-circular perforations, which feature stress concentration points at their vertices, is expected to be more complex and consequential. In previous work by some of the present authors, the residual stress field around two nanoscale polygonal holes under surface tension was investigated using the complex variable method and G–M surface theory [22]. In that earlier study, numerical examples were presented for a triangular hole interacting with a square hole, and the influence of the distance between the two holes on the stress field was analyzed in detail. It was shown that when the normalized distance becomes sufficiently small, strong interaction between the two holes alters the stress distribution, and critical distances could be identified at which the interaction starts to be significant and at which the stress distribution pattern changes. However, that study was limited to a specific polygonal pair (triangular–square) and did not address more general shape combinations or attempt to formulate a unified design principle.

The present work aims to substantially extend this line of research in three primary aspects:

(1) Geometric generalization: The semi-analytical framework is extended from purely polygonal pairs to mixed polygonal–elliptical combinations. The triangular and square approximations represent faceted nano-voids, while the elliptical perforation represents a generic smooth defect, encompassing circular pores and crack-like voids (via its aspect ratio). Such combinations are more representative of realistic pore networks in nanoporous metals and complex composites.
(2) Unified competition mechanism: Through systematic numerical investigations of triangular–elliptical and square–elliptical pairs, the residual stress fields are analyzed with respect to inter-perforation distance and size ratios. It is shown that the stress state can be described by a competition between intrinsic surface tension effects and extrinsic elastic interactions, with a critical distance controlling the transition between a surface-tension-dominated regime and an interaction-dominated regime. This competition mechanism is demonstrated to be robust across different shape combinations, thereby upgrading earlier, shape-specific observations to a general principle.
(3) Design-oriented insights: The numerical results are synthesized into quantitative guidelines for the microstructural design of nanoporous materials. In particular, it is demonstrated that (i) closely spaced perforations lead to severe stress intensification near adjacent vertices, defining likely sites for crack initiation and failure, and (ii) increasing the size of one perforation can, under appropriate configurations, effectively shield a neighboring perforation and reduce the overall stress level. These findings enable a shift from trial-and-error approaches to physics-based, bottom-up design of porous architectures.

The paper is organized as follows. Section 2 presents the theoretical framework, including the G–M surface elasticity model, the complex variable formulation, and the conformal mappings used to represent the triangular, square, and elliptical perforations. Section 3 reports the numerical results for triangular–elliptical and square–elliptical perforation pairs, with a focus on the influence of distance and size on the hoop stress distribution. Section 4 discusses method validation, elucidates the governing competition mechanism, and outlines the design implications and geometric motivation. Section 5 summarizes the main conclusions and highlights potential extensions.

## 2. Theoretical Framework and Solution Method

### 2.1. Problem Statement and Governing Equations

**Figure 1:**
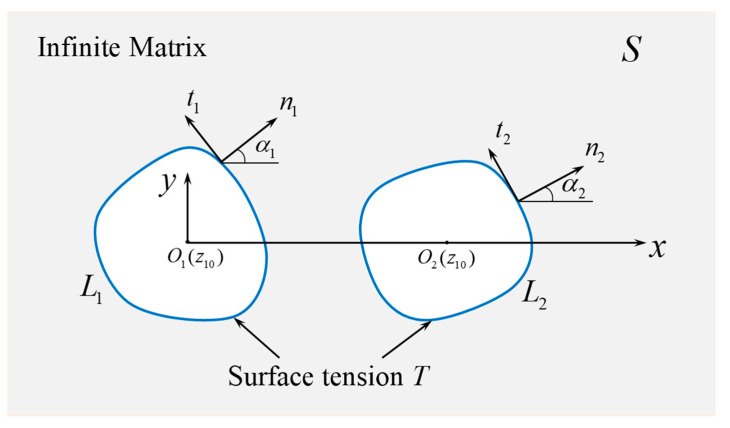
Schematic of the problem: two arbitrarily shaped nano-perforations in an infinite elastic matrix.

Consider an infinite, isotropic, linear elastic matrix under plane strain conditions, containing two arbitrarily shaped nano-perforations, denoted as Perforation 1 and 2, as illustrated in Figure 1. Their boundaries are labeled *L*_1_ and *L*_2_, respectively. A constant residual surface tension *T* acts along each boundary. A Cartesian coordinate system (*xOy*) is established with its origin at the geometric center *O*_1_ of Perforation 1, and the center *O*_2_ of Perforation 2 lies on the positive *x*-axis. The tangent and normal to the perforation boundary are denoted as *t* and *n*, respectively, and α is the angle between the normal and positive *x*-axis.

Following Muskhelishvili’s formalism [23], the displacement ($u_{x}$, $u_{y}$) and stress components ($\sigma_{x}$, $\sigma_{y}$, $\tau_{xy}$) in the matrix can be expressed in the *xOy* coordinate system through two analytic complex potentials φ(z) and ψ(z):

(1) $$\left\{\begin{array}{l} 2\mu (u_{x}+iu_{y})=\kappa \varphi (z)-z\bar{\varphi^{\prime }(z)}-\bar{\psi (z)}\\ \sigma_{x}+\sigma_{y}=2\left[\varphi^{\prime }(z)+\bar{\varphi^{\prime }(z)}\right]\\ \sigma_{y}-\sigma_{x}+2i\tau_{xy}=2\left[\bar{z}\varphi^{\prime \prime }(z)+\psi^{\prime }(z)\right] \end{array}\right.,$$

where μ is the shear modulus, i is the imaginary unit, *z* is the complex variable (*z = x + iy*), and κ=3−4ν for plane strain with *ν* being Poisson’s ratio. From Equation (1), the displacement and stress components in the local normal and tangential coordinate system can be derived via geometrical transformation:

(2) $$\left\{\begin{array}{l} 2\mu (u_{n}+iu_{t})=\left[\kappa \varphi (z)-z\bar{\varphi^{\prime }(z)}-\bar{\psi (z)}\right]e^{-i\alpha }\\ \sigma_{nn}+\sigma_{tt}=2[(\varphi^{\prime }z)+\bar{\left(\varphi^{\prime }z\right)}]\\ \sigma_{tt}-\sigma_{nn}+2i\sigma_{nt}=2[\bar{z}(\varphi^{\prime \prime }z)+(\psi^{\prime }z)]e^{2i\alpha } \end{array}\right..$$

The presence of residual surface tension T introduces a jump in the normal traction across the perforation boundary [11]. For a traction-free perforation, this boundary condition can be integrated to obtain the boundary condition for surface traction:

(3) $$\left[\varphi (z)+z\bar{\varphi^{\prime }(z)}+\bar{\psi_{0}(z)}\right]=Te^{i\alpha_{j}},\;\;z\in L_{j}(j=1,\;2).$$

### 2.2. Conformal Mapping and Solution Procedure

To handle the arbitrary perforation shapes, a conformal mapping function is introduced for each perforation [23]:

(4) $$z=\omega_{j}(\zeta_{j})=z_{j0}+R_{j}(\zeta_{j}+\sum_{k=1}^{M_{j}}m_{jk}\zeta_{j}^{-k}),\;\;\left(j=1,\;2\right)$$

where $z_{j0}$ is the centroid of the *j*-th perforation, $R_{j}$ is a real constant relevant with the perforation size, and $m_{jk}$ are shape-regulated real constants. A truncated form is adopted in Equation (4) for practical purpose. This function maps the exterior of the perforation boundary in the physical *z*-plane to the exterior of the unit circle (denoted as $\Gamma_{j}$) in the parametric $\zeta_{j}$-plane. Points on the unit circle are denoted $\sigma_{j}=\exp(i\theta_{j})$. With conformal mapping, we have

(5) eiαj=ζj ωj′ζjρj ωj′ζj,  e2iαj=ζj 2ωj′ζjρj 2ωj′ζj¯.

The matrix domain is the intersection of the exteriors of the two perforations. Using superposition, the complex potentials in the matrix are constructed as:

(6) $$\left\{\begin{array}{l} \varphi \left(z\right)=\sum_{j=1}^{2}\varphi_{j}\left(z\right)=\sum_{j=1}^{2}\varphi_{j}\left(\omega_{j}\left(\zeta_{j}\right)\right)=\sum_{j=1}^{2}\varphi_{j}\left(\zeta_{j}\right)\;\;\;\\ \psi \left(z\right)=\sum_{j=1}^{2}\psi_{j}\left(z\right)=\sum_{j=1}^{2}\psi_{j}\left(\omega_{j}\left(\zeta_{j}\right)\right)=\sum_{j=1}^{2}\psi_{j}\left(\zeta_{j}\right) \end{array}\right.$$

where $\varphi_{j}\left(\zeta_{j}\right)$ and $\psi_{j}\left(\zeta_{j}\right)$ are analytic complex functions outside the unit circle; thus they can be expanded into Laurent series [24]:

(7) $$\varphi_{j}\left(\zeta_{j}\right)=\sum_{k=1}^{N}a_{j}^{\left(k\right)}\zeta_{j}^{-k},\;\;\psi_{j}\left(\zeta_{j}\right)=\frac{\sum_{k=1}^{N}b_{j}^{\left(k\right)}\zeta_{j}^{-k}}{{\omega_{j}}^{\prime }\left(\zeta_{j}\right)},\;\;\;\;\;\;\;\left(j=1,\;2\right)$$

where $a_{j}^{\left(k\right)}$ and $b_{j}^{\left(k\right)}$ are unknown complex coefficients.

Inserting Equation (7) into the stress boundary condition (3) at perforation 1 gives:

(8) $$\sum_{k=1}^{N}\left[a_{1}^{\left(k\right)}\sigma_{1}^{-k}+a_{2}^{\left(k\right)}\zeta_{2}^{-k}+\bar{a_{1}^{\left(k\right)}}\frac{\omega_{1}\left(\sigma_{1}\right)}{\bar{{\omega_{1}}^{\prime }\left(\sigma_{1}\right)}}(-k)\sigma_{1}^{k+1}+\bar{a_{2}^{\left(k\right)}}\frac{\omega_{1}\left(\sigma_{1}\right)}{\bar{{\omega_{2}}^{\prime }\left(\zeta_{2}\right)}}(-k)\bar{\zeta_{2}^{-k-1}}+\frac{\bar{b_{1}^{\left(k\right)}}\sigma_{1}^{k}}{\bar{{\omega_{1}}^{\prime }\left(\sigma_{1}\right)}}+\frac{\bar{b_{2}^{\left(k\right)}}\bar{\zeta_{2}^{-k}}}{\bar{{\omega_{2}}^{\prime }\left(\zeta_{2}\right)}}\right]=T\sigma_{1}\frac{{\omega_{1}}^{\prime }\left(\sigma_{1}\right)}{\left|{\omega_{1}}^{\prime }\left(\sigma_{1}\right)\right|},$$

in which the parametric coordinate $\zeta_{2}$ is calculated through

(9) ζ2=ω2ω1σ1−1,     ζ2>1.

Therefore, all terms in the boundary condition (8) are functions of $\sigma_{1}$. Similarly, inserting Equation (7) into the stress boundary condition (3) at perforation 2 gives:

(10) $$\sum_{k=1}^{N}\left[a_{1}^{\left(k\right)}\zeta_{1}^{-k}+a_{2}^{\left(k\right)}\sigma_{2}^{-k}+\bar{a_{1}^{\left(k\right)}}\frac{\omega_{2}\left(\sigma_{2}\right)}{\bar{{\omega_{1}}^{\prime }\left(\zeta_{1}\right)}}(-k)\bar{\zeta_{1}^{-k-1}}+\bar{a_{2}^{\left(k\right)}}\frac{\omega_{2}\left(\sigma_{2}\right)}{\bar{{\omega_{2}}^{\prime }\left(\sigma_{2}\right)}}(-k)\sigma_{2}^{k+1}+\frac{\bar{b_{1}^{\left(k\right)}}\bar{\zeta_{1}^{-k}}}{\bar{{\omega_{1}}^{\prime }\left(\zeta_{1}\right)}}+\frac{\bar{b_{2}^{\left(k\right)}}\sigma_{2}^{k}}{\bar{{\omega_{2}}^{\prime }\left(\sigma_{2}\right)}}\right]=T\sigma_{2}\frac{{\omega_{2}}^{\prime }\left(\sigma_{2}\right)}{\left|{\omega_{2}}^{\prime }\left(\sigma_{2}\right)\right|},$$

in which the parametric coordinate $\zeta_{1}$ is calculated through

(11) ζ1=ω1ω2σ2−1,     ζ1>1.

Therefore, all terms in the boundary condition (10) are functions of $\sigma_{2}$. These equations can be solved by using Fourier series expansion methods. After expanding every term in the boundary conditions (8) or (10) into Fourier series in terms of $\sigma_{1}$ or $\sigma_{2}$, one can obtain a system of linear equations for the unknown coefficients $a_{j}^{\left(k\right)}$ and $b_{j}^{\left(k\right)}$ by comparing coefficients for like terms of $\sigma_{j}^{\left(k\right)}$. This system can be solved numerically using computational software. Once the coefficients are determined, the stress components, particularly the hoop stress, can be calculated throughout the domain using Equation (2).

## 3. Numerical Results

In this section, the numerical results for the residual stress field around two interacting nano-perforations are presented. The calculations are performed for triangular–elliptical and square–elliptical perforation pairs, with particular emphasis on the hoop (circumferential) stress along the perforation boundaries. The influence of inter-perforation distance and relative perforation size is examined to identify stress concentration patterns, interaction regimes, and potential shielding effects. The conformal mapping functions for the considered shapes are:

(12) $$\left\{\begin{array}{l} z-z_{0}=\omega (\zeta )=R(\zeta +0.5\zeta^{-1})\;\;\;\;\;\;\;\;\;\;\mathrm{Elliptical}\;\mathrm{perforation}\;\left(\mathrm{eccentricity}\;0.5\right)\\ z-z_{0}=\omega (\zeta )=R(\zeta +\frac{\zeta^{-2}}{3})\;\;\;\;\;\;\;\;\;\;\;\;\mathrm{Approximately}\;\mathrm{triangular}\;\mathrm{perforation}\\ z-z_{0}=\omega (\zeta )=R(\zeta +\frac{\zeta^{-3}}{6})\;\;\;\;\;\;\;\;\;\;\;\;\mathrm{Approximately}\;\mathrm{square}\;\mathrm{perforation}\;\;\; \end{array}\right.$$

### 3.1. Interaction Between a Triangular and an Elliptical Perforation

To begin, the interaction between a faceted triangular perforation and a smooth elliptical perforation is considered. The triangular hole represents a sharp, crystallographically faceted void, while the elliptical hole represents a generic pore or a micro-crack, depending on its aspect ratio. This configuration mimics situations where sharp grain boundary cavities coexist with smoother defects in nanoporous metals or etched thin films. The goal is to quantify how the presence and proximity of the elliptical perforation modify the stress concentration at the vertices of the triangular perforation.

**Figure 2:**
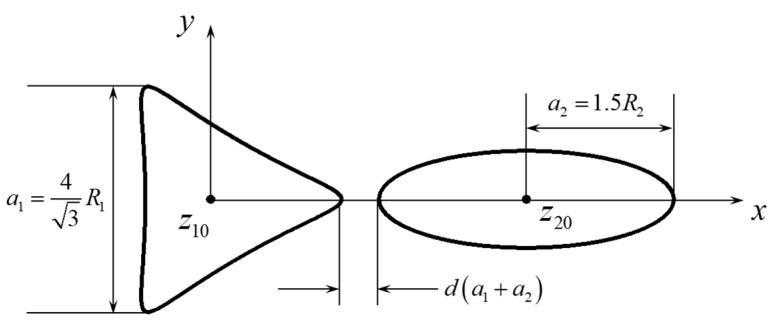
Configuration of the triangular and elliptical perforations. $R_{1}$ and $R_{2}$ are two constants that control the sizes of the two perforations.

The configuration of the perforations is shown in Figure 2. The side length of the triangle is related to the constant *R* in the conformal mapping as $a_{1}=\left(4/\sqrt{3}\right)R_{1}$, and the major semi-axis of the ellipse is related to the constant *R* in the conformal mapping as $a_{2}=1.5R_{2}$. The distance between the centers is defined by a parameter *d* normalized by the perforation sizes (see Equation (13)), and the relative size is defined as $l=R_{2}/R_{1}$. The origin is put at the centroid of the triangular perforation; thus the centroid coordinate of the elliptical perforation is

(13) $$\begin{array}{l} z_{20}=\frac{\sqrt{3}}{3}a_{1}+d\left(a_{1}+a_{2}\right)+a_{2}\\ \;\;\;=\frac{4}{3}R_{1}+d\left(\frac{4}{\sqrt{3}}R_{1}+1.5R_{2}\right)+1.5R_{2}. \end{array}$$

The residual stress field is determined for a range of *d* values (0.1, 0.5, 3, 100), and the normalized hoop stress $\sigma_{\mathrm{tt}}T/R$ along the boundary of the triangular hole is evaluated.

**Figure 3:**
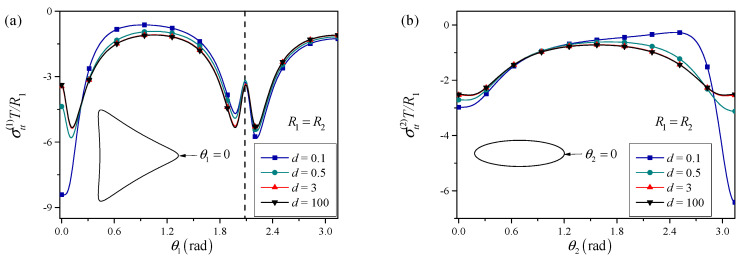
Influence of the distance between the two perforations on the hoop stress distributions around the (**a**) triangular perforation and (**b**) elliptical perforation. The dash line indicates the position of the maximum curvature point along the perforation boundary, where a local minimum of the hoop stress is achieved.

Figure 3 shows that for large normalized distances, the presence of the elliptical perforation has only a minor influence on the stress distribution around the triangle. The hoop stress profile along the triangular boundary closely matches that of an isolated triangular nano-hole under surface tension, exhibiting moderate stress concentration near the vertices due to geometric sharpness and surface effects. As the distance decreases, the stress field becomes progressively more perturbed. For intermediate distances (e.g., d≈3), noticeable amplification of hoop stress is observed at the vertex of the triangle that faces the elliptical perforation. When the distance becomes small (e.g., d≤0.5), a strong interaction regime is reached. The maximum hoop stress at the facing vertex increases dramatically compared to the isolated case, indicating a significant risk of crack initiation or local yielding at this location. This behavior is consistent with the notion that below a certain critical distance, the elastic interaction between perforations dominates the stress state, overriding the surface-tension-induced smoothing.

**Figure 4:**
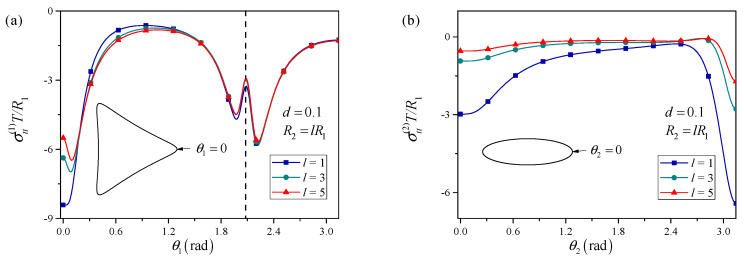
Influence of the relative size between the two adjacent perforations on the hoop stress distributions around the (**a**) triangular perforation and (**b**) elliptical perforation.

The effect of the elliptical hole size is also examined in Figure 4. When the elliptical perforation is enlarged while keeping the triangular hole size fixed, it is observed that, for certain ranges of *d*, the stress at the triangular vertices can be reduced compared to the case with two holes of similar size. This indicates a shielding effect, whereby a sufficiently large neighboring perforation redistributes the stress in such a way that the smaller perforation experiences a lower local stress concentration. The magnitude of this shielding effect depends on both the size ratio and the distance.

### 3.2. Interaction Between a Square and an Elliptical Perforation

The analysis is next extended to the interaction between a square perforation and an elliptical perforation. The square hole represents a faceted void with approximate 90° corners, which is another common morphology in crystalline or lithographically patterned materials. This case is used to examine whether the interaction features observed in the triangular–elliptical configuration persist when the polygonal shape is changed and to explore the influence of shape symmetry on stress localization.

**Figure 5:**
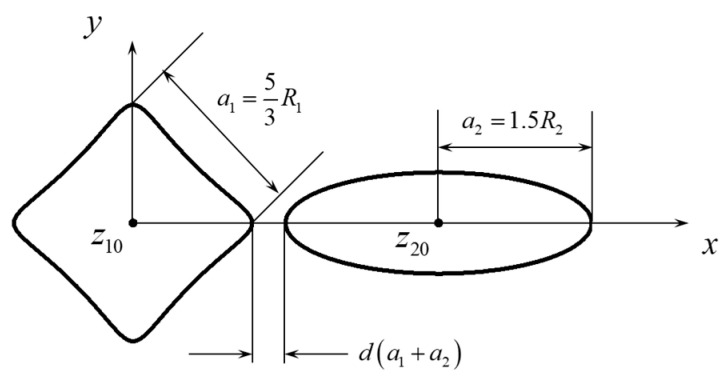
Configuration of the square and elliptical perforations. $R_{1}$ and $R_{2}$ are two constants that control the sizes of the two perforations.

The configuration is shown in Figure 5. The side length of the square is $a_{1}=\left(5/3\right)R_{1}$. The origin is put at the centroid of the triangular perforation; thus the centroid coordinate of the elliptical perforation is

(14) $$\begin{array}{l} z_{20}=\frac{\sqrt{2}}{2}a_{1}+d\left(a_{1}+a_{2}\right)+a_{2}\\ \;\;\;=\frac{5\sqrt{2}}{6}R_{1}+d\left(\frac{5}{3}R_{1}+1.5R_{2}\right)+1.5R_{2} \end{array}$$

The normalized distance *d* between their centers is varied, and the hoop stress distribution along the square boundary is computed.

**Figure 6:**
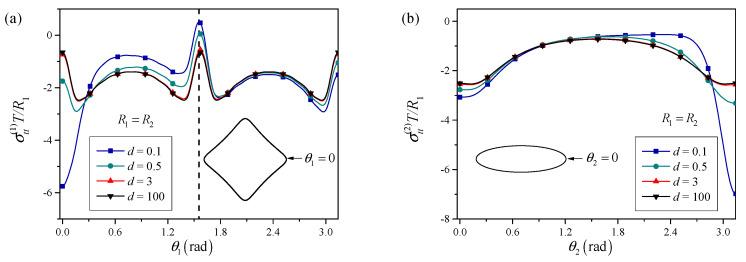
Influence of the distance between the two perforations on the hoop stress distributions around the (**a**) square perforation and (**b**) elliptical perforation.

The influence of inter-perforation distance is first examined. Figure 6 shows that at large distances (*d* = 100), the stress distribution around the square hole resembles that of an isolated nano-square under surface tension. The hoop stress is higher at the corners than along the flat edges, reflecting classical stress concentration at sharp corners. The hoop stress also attains a local minimum at the corners, which is the feature of surface tension effect. As the distance is reduced to intermediate values, the influence of the elliptical perforation becomes apparent. The corner of the square facing the elliptical hole exhibits increased hoop stress, whereas the opposite corner may experience a slight reduction, indicating a non-uniform redistribution of stresses. When the distance becomes small (d≤0.5), a strong interaction regime develops. The maximum hoop stress is found at the square corner closest to the elliptical perforation, with values significantly exceeding those in the single-hole or large-distance cases. The local stress field in the ligament between the two holes is highly intensified, marking this region as a critical site for damage initiation.

**Figure 7:**
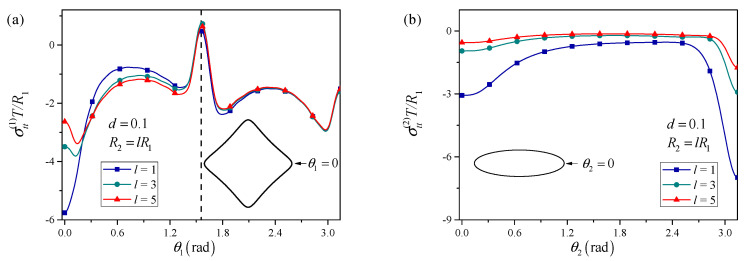
Influence of the relative size between the two adjacent perforations on the hoop stress distributions around the (**a**) square perforation and (**b**) elliptical perforation.

The effect of the elliptical hole size is again investigated in Figure 7. For larger elliptical perforations, a shielding effect similar to that observed in the triangular–elliptical case can arise. When the elliptical hole is sufficiently large, the stress concentration at the nearest square corner may be reduced compared to a configuration with two similarly sized holes. This indicates that a large perforation can, under appropriate conditions, relieve the stress at a neighboring sharper perforation by redistributing the stress flow through the matrix. The extent of this shielding depends on the relative sizes, the shape of the elliptical hole, and the spacing.

## 4. Discussions

### 4.1. Method Validation and Relation to Previous Works

The reliability of the semi-analytical framework employed in this study is supported by both limiting-case consistency and comparison with previous analytical results.

First, in the limit of infinite separation between perforations (d→∞), the interaction between holes vanishes. In this limit, the computed stress fields around each perforation are reduced to those of isolated nano-holes with surface effects, for which analytical or semi-analytical solutions have been reported in the literature [11,15]. This convergence confirms that the present formulation correctly captures the decay of interaction effects at large distances.

Second, the present work builds upon an established complex variable and G–M surface elasticity framework previously used by some of the authors to study the interaction between a triangular and a square nano-hole [22]. In that earlier study, the method was applied to a specific polygonal–polygonal pair, and critical distances were identified at which interaction becomes significant and at which the stress distribution pattern changes. The current extension to polygonal–elliptical combinations maintains the same mathematical foundation but requires additional conformal mappings to represent elliptical boundaries. The qualitative behavior observed in the new configurations—namely, the onset of interaction at a critical distance and the alteration of stress patterns at shorter distances—is consistent with the trends reported previously, supporting the validity of the present approach.

The combination of limiting-case checks and consistency with earlier verified results provides a strong basis for confidence in the semi-analytical solutions obtained here.

### 4.2. Governing Competition Mechanism

The consistent findings across different shape pairs indicates a unified model for the stress state in nanoscale porous materials. The hoop stress at any point, particularly at critical locations like corners, is co-determined by the intrinsic surface tension effect and the extrinsic elastic interaction.

- Intrinsic Factor (Surface Tension): This effect scales with the local curvature and is a property of the individual perforation. It dictates the stress distribution when perforations are widely spaced.
- Extrinsic Factor (Perforation Interaction): This effect scales with the inverse of the distance between perforation boundaries and depends on their relative orientation and size. It dictates the stress redistribution when perforations are in close proximity.

The parameter *d* effectively controls which factor is dominant. Our results establish that the critical threshold for this transition lies at d≈3. For *d >* 3, the intrinsic effect prevails, and single-perforation models are sufficient. For *d <* 3, the extrinsic interaction effect becomes dominant, and a full interaction analysis is mandatory for accurate stress prediction.

This competition framework has profound implications for material design. It suggests that the mechanical performance of a nanoporous material is not an intrinsic property but an emergent one, dependent on the collective configuration of the perforation network. By mapping this competition, this study moves from a descriptive understanding of stress concentrations to a predictive capability for managing them through microstructural design.

### 4.3. Geometric Motivation and Design Implications

The choice of triangular, square, and elliptical perforations in this study is motivated by the desire to bridge idealized models with realistic microstructures. Triangular and square perforations serve as simplified models of faceted voids and grain-boundary cavities that form along specific crystallographic orientations during material processing or service. Elliptical perforations represent a versatile class of smooth defects, including circular pores and crack-like voids, depending on their aspect ratios. This makes ellipses an effective surrogate for a wide variety of smooth defects.

By combining faceted and smooth shapes, the configurations studied here capture typical interactions between sharp and blunt defects that arise in nanoporous metals, porous catalysts, functional composites, and MEMS/NEMS devices.

## 5. Conclusions and Implications for Material Design

This study successfully demonstrates the use of a semi-analytical framework for simulating the complex residual stress fields in nanoscale materials containing multiple perforations. The key mechanical insights, directly applicable to the design of advanced porous materials and perforated structures, are as follows:

(a) Governing competition mechanism: The residual stress field is governed by a competition between intrinsic surface tension effects and extrinsic elastic interactions between perforations. The normalized inter-perforation distance *d* serves as the control parameter, with a critical threshold of d≈3 marking the transition from a surface-tension-dominated regime to an interaction-dominated regime.
(b) Geometric proximity is critical and synergistic: The distance between perforations is a primary factor controlling stress concentration. Densely packed perforated structures are prone to severe stress intensification at regions where perforations are in close proximity, defining likely sites for crack initiation and mechanical failure. Our analysis reveals that this is due to a synergistic superposition of stress fields in the inter-perforation ligament, establishing the perforation pair as the critical failure unit.
(c) Size distribution as a design tool and the “shielding effect”: The relative size of adjacent perforations can be leveraged to manage stress. A larger perforation can shield a smaller, adjacent perforation by reducing the stress concentration at the nearby vertex. This is mechanistically explained as the larger perforation’s stress field dominating and beneficially influencing the inter-perforation region.
(d) Universality: The fundamental phenomena of stress intensification with proximity and shielding with size disparity are robust across different shape combinations (triangular/elliptical, square/elliptical). This universality makes these findings widely applicable for the design of various nanoscale porous and perforated materials.

These conclusions provide quantitative, physics-based guidelines for the microstructural design of nanoporous materials. By consciously tailoring the spatial distribution, size, and shape of perforations, materials scientists can move beyond trial-and-error and towards the rational design of materials with optimized mechanical reliability for applications in catalysis, filtration, and lightweight structural components.

## Data Availability

The original contributions presented in this study are included in the article. Further inquiries can be directed to the corresponding authors.
